# Accuracy of Inferior Vena Cava Ultrasound for Predicting Dehydration in Children with Acute Diarrhea in Resource-Limited Settings

**DOI:** 10.1371/journal.pone.0146859

**Published:** 2016-01-14

**Authors:** Payal Modi, Justin Glavis-Bloom, Sabiha Nasrin, Allysia Guy, Erika P. Chowa, Nathan Dvor, Daniel A. Dworkis, Michael Oh, David M. Silvestri, Stephen Strasberg, Soham Rege, Vicki E. Noble, Nur H. Alam, Adam C. Levine

**Affiliations:** 1 Department of Emergency Medicine, The Warren Alpert Medical School of Brown University, Providence, Rhode Island, United States of America; 2 International Centre for Diarrhoeal Disease Research, Mohakhali, Dhaka, Bangladesh; 3 Department of Emergency Medicine, Lincoln Medical and Mental Health Center, Bronx, New York, United States of America; 4 Department of Emergency Medicine, Harvard Medical School, Boston, Massachusetts, United States of America; Centre Hospitalier Universitaire Vaudois, FRANCE

## Abstract

**Introduction:**

Although dehydration from diarrhea is a leading cause of morbidity and mortality in children under five, existing methods of assessing dehydration status in children have limited accuracy.

**Objective:**

To assess the accuracy of point-of-care ultrasound measurement of the aorta-to-IVC ratio as a predictor of dehydration in children.

**Methods:**

A prospective cohort study of children under five years with acute diarrhea was conducted in the rehydration unit of the International Centre for Diarrhoeal Disease Research, Bangladesh (icddr,b). Ultrasound measurements of aorta-to-IVC ratio and dehydrated weight were obtained on patient arrival. Percent weight change was monitored during rehydration to classify children as having “some dehydration” with weight change 3–9% or “severe dehydration” with weight change > 9%. Logistic regression analysis and Receiver-Operator Characteristic (ROC) curves were used to evaluate the accuracy of aorta-to-IVC ratio as a predictor of dehydration severity.

**Results:**

850 children were enrolled, of which 771 were included in the final analysis. Aorta to IVC ratio was a significant predictor of the percent dehydration in children with acute diarrhea, with each 1-point increase in the aorta to IVC ratio predicting a 1.1% increase in the percent dehydration of the child. However, the area under the ROC curve (0.60), sensitivity (67%), and specificity (49%), for predicting severe dehydration were all poor.

**Conclusions:**

Point-of-care ultrasound of the aorta-to-IVC ratio was statistically associated with volume status, but was not accurate enough to be used as an independent screening tool for dehydration in children under five years presenting with acute diarrhea in a resource-limited setting.

## Introduction

Each year, children under 5 years of age worldwide experience 1.7 billion diarrheal episodes, leading to 124 million outpatient visits and 9 million hospitalizations [1,2]. Of these, approximately 36 million cases each year progress to severe disease, accounting for over 700,000 deaths annually [3]. Accurately assessing dehydration status is critical to providing targeted treatment and preventing mortality and morbidity. Children with mild to moderate dehydration do better when treated with oral rehydration solution (ORS) alone while those with severe disease require intravenous fluids (IVF) to limit life-threatening complications [4]. However, assessment of dehydration status can be challenging, as no individual clinical sign, symptom or laboratory test has demonstrated adequate sensitivity, specificity, and reliability for detecting dehydration in children [5,6].

Point-of-care ultrasound has become a popular tool for evaluating a variety of obstetric and non-obstetric conditions in rural hospitals and refugee camps in low- and middle-income countries [7,8]. Its relative safety, cost-effectiveness, portability, versatility, and ease of use make it an ideal diagnostic tool in these settings. In addition to its many other uses, ultrasound has recently emerged as a potential tool for assessing volume status in children and adults. Several recent studies have identified ultrasound of the aorta-to-inferior vena cava (IVC) ratio as a promising means to identify dehydration in children, though all have been relatively small in size with few children presenting with severe dehydration. Consequently, a recent systematic review did not find sufficient evidence to encourage the use of ultrasound for diagnosing dehydration in children [6].

This study aims to assess the accuracy of aorta-to-IVC ratio as a predictor of severe dehydration in children presenting with acute diarrhea in a resource-limited setting, as well as the effect of volume resuscitation on IVC and aorta diameter in a subset of children.

## Materials & Methods

### Study Design

This research was conducted as part of the Dehydration: Assessing Kids Accurately (DHAKA) study. The DHAKA study prospectively enrolled a random sample of children with acute diarrhea presenting to the rehydration unit of the International Centre for Diarrhoeal Disease Research, Bangladesh (icddr,b) between February and June, 2014. Ethical approval for the study was obtained from the Lifespan (Rhode Island Hospital) Institutional Review Board and the icddr,b Ethical Review Committee. The study was registered with ClinicalTrials.gov Identifier: NCT02007733.

### Setting and participant selection

Overall, icddr,b serves a catchment area of 17 million people and provides treatment for approximately 144,000 patients yearly, over half of whom are children [9]. All children under five years presenting to the rehydration (short stay) unit at icddr,b were eligible for enrollment. Study staff randomly selected children for screening on arrival 24 hours per day, 7 days per week by pulling blue (selected) or white (not selected) marbles from a blind pouch. Once selected, patients were assessed for any pre-defined exclusion criteria, included having less than three loose stools per day, a diarrheal illness lasting longer than 14 days, a clear alternative diagnosis to gastroenteritis on arrival, or having been previously enrolled in the study. For patients who did not meet any of these exclusion criteria, research staff approached the patient’s parent/guardian, explained the risks and benefits of the study in the local language, Bengali, and obtained written or verbal consent, depending on the parent/guardian’s level of literacy.

### Staff Training

Research staff included eight general practice nurses with four to six years of clinical experience. All underwent a seven-day training course consisting of both theoretical knowledge and practical experience.

A board-certified emergency physician with fellowship training in emergency ultrasound conducted the ultrasound portion of the training. On the first two days of the training, research nurses were given two and half hours of didactic lectures and four hours of hands-on practice acquiring images of the aorta and IVC on adult models. Information taught during the didactic sessions included machine maintenance, basic functioning, patient data management, point of care ultrasound technique, and identification of abdominal structures important for the study. A practical quiz was conducted at the end of this period, during which research nurses were observed and evaluated on their ultrasound proficiency; ability to set up the ultrasound machine, enter patient identifying data, save images, and perform measurements of the IVC and the aorta.

On the third and fourth day of training, research nurses attended two sessions dedicated to identifying pitfalls. Together the group analyzed the quality of images of the aorta and IVC and discussed how to improve image quality and avoid common mistakes. Splenic vein, portal vein, and vertebral shadow identification were specifically discussed as the most common pitfalls when identifying and measuring the IVC and the aorta.

On the fifth through seventh day of training, each trainee had at least 6 hours of hands-on practice obtaining images of the aorta and IVC on children in the rehydration unit. Each trainee performed at least 25 ultrasounds under direct supervision. Total sonographic training time for each nurse was about 20 hours, including didactics and practical sessions.

After the study began, two ultrasound-fellowship trained physicians randomly reviewed approximately five ultrasounds performed by each nurse at one week, one month and two months into the study for a total of 100 ultrasound reviews over the course of the study. Nurses were directly provided feedback in real time on technique, identification, and accuracy of vessel measurement by the ultrasound-fellowship trained experts.

### Methods of Measurements

Immediately after consent was obtained, children were undressed and weighed to the nearest 100 grams using an electronic scale in order to obtain a baseline “dry” weight. Ultrasound measurements of the aorta and IVC diameters were then obtained by research nurses using a SonoSite M-Turbo (SonoSite Inc, Bothell, Wash) ultrasound machine and the P21 5–1 MHz phased array transducer. For the full ultrasound protocol, see S1 File. Briefly, the child was laid flat and the research nurse placed the ultrasound probe in the transverse position (probe marker facing to the patient’s right) just inferior to the xyphoid process. The aorta was identified above the vertebral shadow, and the IVC was identified to its screen left, closer to the probe marker. Measurements were then taken in the anterior-posterior orientation from inner wall to inner wall for both the aorta and IVC at their maximum diameter, saving all images and measurements. Study nurses subjectively assessed both the quality of images obtained and overall confidence in their measurements for each patient.

Patients were then rehydrated according to standard hospital protocols. During rehydration, patients were weighed every eight hours until they achieved a stable weight. Stable weights were obtained for each enrolled patient by averaging the two highest consecutive weight measurements that differed by less than 2%, as described in the literature [10]. Generally, dehydrated children rapidly gain weight with rehydration until they achieve their pre-illness weight, or stable weight, at which point their weight will plateau as their kidneys diurese excess fluid.

After rehydration to a stable weight, a random sample of children had a repeat ultrasound performed, with measurements of the aorta and IVC diameter obtained using the same methodology as for the initial exam. Both the initial and repeat aorta-to-IVC ratio were calculated for each child by dividing the maximal aorta diameter in centimeters by the maximal IVC diameter in centimeters. Given that both IVC and aorta size would be expected to increase as the child grows, aorta-to-IVC ratio was used instead of IVC diameter alone as a predictor of dehydration in children, as has been previously described in the literature [11,12].

Finally, research nurses obtained basic historical and demographic data for each child from their parent/guardian. Malnutrition was assessed for all children enrolled by measuring the mid-upper arm circumference (MUAC) to the nearest millimeter using a standard measuring tape. MUAC < 115 mm was considered severe malnutrition while a MUAC of 115–125 mm was considered moderate malnutrition [13].

### Outcome Measures

For each patient with a valid stable weight, we calculated the percent weight change with rehydration, our proxy for percent dehydration, using the following formula:

Percent Dehydration ≈ ((Stable Weight–Admission Weight) / Stable Weight) * 100

If children did not achieve a stable weight prior to discharge, their parent/guardian was called daily by mobile phone until the child’s diarrhea had completely resolved, at which point they were asked to return to the rehydration unit for a post-illness weight. The post-illness weight was then used instead of the stable weight in the formula above to calculate the percent dehydration. Based on the percent dehydration, children were categorized as having no dehydration (<3%), some dehydration (3–9%), and severe dehydration (>9%). Children who lost significant weight (greater than 3% of their admit weight) during their stay in the rehydration unit, suggesting inadequate hydration in the face of ongoing diarrhea or an error in data collection, were excluded from analysis.

### Primary Data Analysis

Baseline historical, demographic, and nutritional data were summarized for all children enrolled in our study and compared for those children included and excluded from analysis using the chi-square and equality of medians test as appropriate. Descriptive statistics were used to calculate the median percent dehydration and the proportion of children with no dehydration, some dehydration, and severe dehydration. Ultrasound operational variables, including timing of exams, image quality, and nurse confidence, were summarized. Quality assurance measures including the correct identification and measurement of aorta and IVC were also summarized.

A standard linear regression model was used to assess the association between aorta-to-IVC ratio and percent dehydration, controlling for patient age, sex, and malnutrition status (MUAC). Separate logistic regression models were employed to assess aorta-to-IVC ratio as an independent predictor for the outcome of some dehydration and severe dehydration, respectively, using odds ratios. The area under the Receiver-Operator Characteristic (ROC) curve was used to assess the discrimination of aorta-to-IVC ratio for the outcomes of some dehydration and severe dehydration. Test characteristics, including sensitivity, specificity and positive and negative likelihood ratios were calculated for the outcome of severe dehydration.

For children randomly selected to have a repeat ultrasound after rehydration, the percent change in IVC and aorta diameter between the initial and repeat ultrasound was calculated. We used the student’s t test to assess whether the percent change in IVC size was statistically greater than the change in aorta size, and also to determine whether change in IVC size was greater in patients with severe dehydration compared to those without severe dehydration. All statistical analyses were performed using STATA 12.0 (STATA Corp, College Station, Texas).

## Results

### Enrollment, Baseline Characteristics, and Outcomes

**Fig 1:**
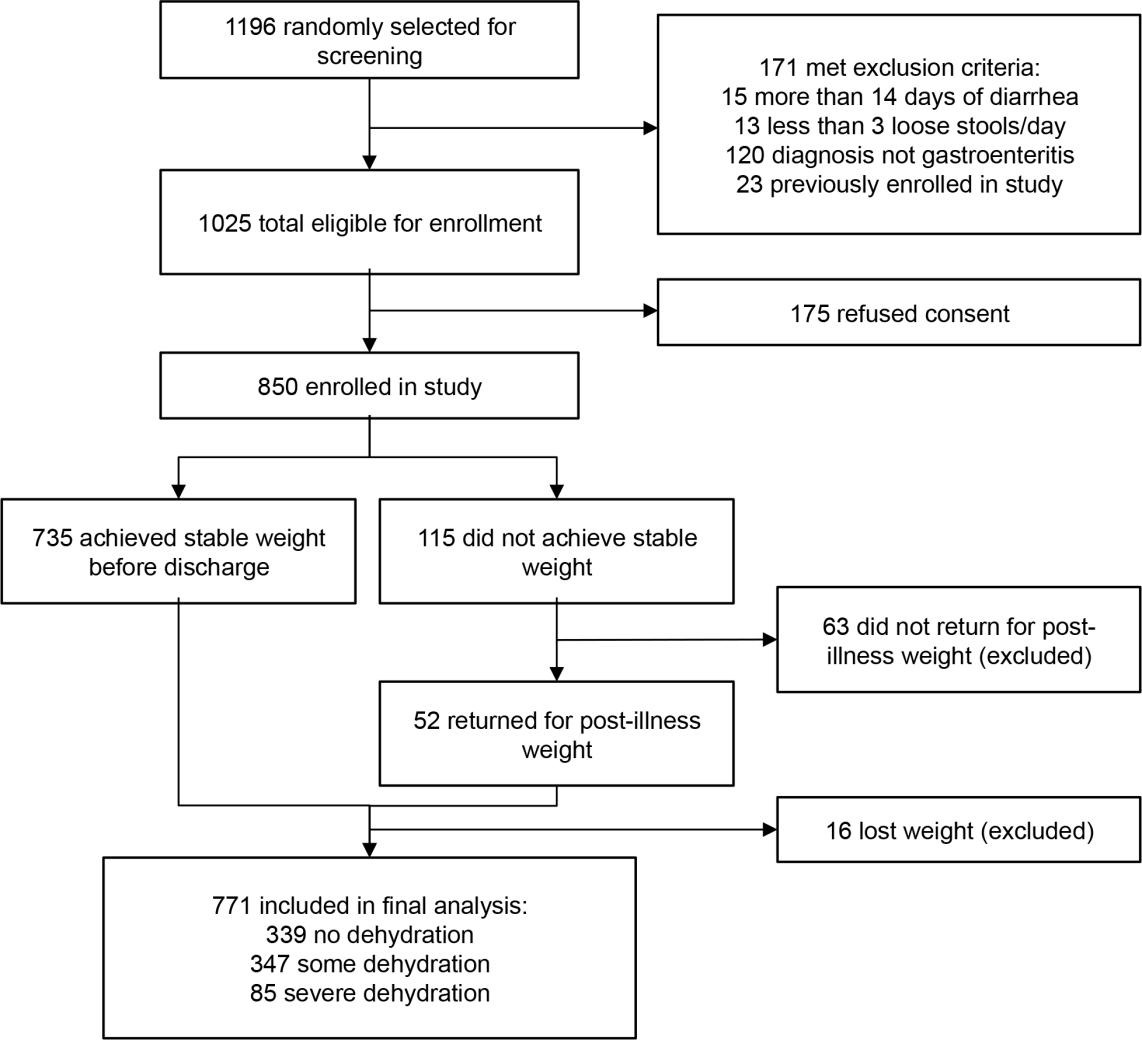
Patient enrollment.

Of 850 children enrolled, 771 had complete outcome data available for analysis (Fig 1). There were no significant differences in baseline demographic, historical, or anthropometric characteristics between those included and excluded from analysis (Table 1). Among the 771 patients included in analysis, the median percent dehydration on arrival in study subjects, as measured by the percent weight change with rehydration, was 4% (IQR: 1%, 7%). Eighty five (11%) children were classified with severe dehydration, 347 (45%) with some dehydration, and 339 (44%) with no dehydration. Overall, median time from patient arrival to achievement of stable weight was 14 hours (IQR = 11, 19) for the 735 patients who achieved a stable weight prior to discharge.

**Table 1:** Baseline data.

	Included in final analysis (n = 771)	Excluded, lost weight (n = 16)	Excluded, no final weight (n = 63)	p-value
**Age in months, median (IQR)**	15 (9–29)	18 (13–29)	22 (12–36)	0.07*
Gender				0.84†
** Female, n (%)**	336 (44)	6 (38)	26 (41)	
** Male, n (%)**	435 (56)	10 (62)	37 (59)	
Home district				0.99†
** Urban (Dhaka), n (%)**	478 (62)	14 (88)	45 (71)	
** Rural/Suburban, n (%)**	293 (38)	2 (12)	18 (29)	
**Nutritional status (MUAC)**				0.30†
** No acute malnutrition, n (%)**	614 (80)	16 (100)	53 (84)	
** Moderate acute malnutrition (MAM), n (%)**	121 (16)	0 (0)	7 (11)	
** Severe acute malnutrition (SAM), n (%)**	35 (4)	0 (0)	3 (5)	
**Days of diarrhea prior to arrival, median (IQR)**	2 (1–4)	2 (1.5–3.5)	2 (1–3)	0.13*
**Loose stools in past 24 hours, median (IQR)**	15 (10–20)	15 (11–20)	15 (10–20)	0.79*
**Diarrhea type**				0.69†
** Watery, n (%)**	448 (58)	12 (75)	36 (57)	
** Rice-Water, n (%)**	317 (41)	4 (25)	27 (43)	
** Bloody, n (%)**	4 (1)	0 (0)	0 (0)	

*Equality of Medians

†Chi Square

### Ultrasound Investigation

Median time from patient arrival in the rehydration unit to performance of the ultrasound exam was 10 minutes (IQR: 7, 13). Among all 771 arrival ultrasounds, 617 (80%) were deemed by research nurses to have good image quality, while 133 (17%) were interpretable but of poor quality, and 21 (3%) were not interpretable. Of the 750 ultrasound exams considered interpretable, research nurses marked their confidence level in their ultrasound performance as low in 115 (15%) scans, moderate in 232 (31%) and high in 403 (54%) scans. Median time for nurses to perform the ultrasound exam was 6 minutes (IQR: 4, 8) (Table 2).

**Table 2:** Ultrasound results.

	Study ultrasounds
Nurse-rated ultrasound image quality, n (%)	
** Good**	617 (80)
** Poor**	133 (17)
** Uninterpretable**	21 (3)
**Nurse-rated ultrasound confidence, n (%)**	
** High**	403 (54)
** Moderate**	232 (31)
** Low**	115 (15)
**Physician quality assurance review of 100 exams, n (%)**	
** Aorta correctly identified**	100 (100)
** Aorta correctly measured**	99 (99)
** **IVC correctly identified	76 (76)
** IVC correctly measured**	75 (99)
** IVC not correctly identified**	11 (11)
** Unclear whether IVC was correctly identified**	13 (13)

Two ultrasound-fellowship trained physicians performed a quality assurance review of video and still images from 100 randomly selected ultrasound exams at regularly spaced intervals over the course of the study. Per their review, research nurses correctly identified the aorta in 100% of subjects and the IVC in at least 76% of subjects; nurses correctly measured the aorta and IVC diameters in 99% of subjects for whom the correct vessel was identified. In 11% of cases, research nurses clearly misidentified another structure as the IVC, including the splenic vein, portal vein, and symmetric shadowing above the vertebral body. In 13% of cases, it was unclear whether the research nurses had correctly identified the IVC (Table 2).

### Prediction of severe dehydration

Linear regression analysis of aorta-to-IVC ratio was performed for the 750 subjects with interpretable ultrasound exams on arrival. Aorta to IVC ratio predicted the percent dehydration in children with acute diarrhea, with each 1 point increase in the aorta to IVC ratio predicting a 1.1% increase in the percent dehydration of the child (95% CI: 0.7%, 1.5%). After controlling for age, gender, and malnutrition status (MUAC), aorta-to-IVC ratio independently predicted the percent dehydration in children with acute diarrhea, with each 1 point increase in the aorta-to-IVC ratio predicting a 0.8% increase in the percent dehydration of the child (95% CI: 0.4%, 1.2%).

In logistic regression analysis, aorta-to-IVC ratio predicted both some dehydration and severe dehydration. Each 1-point increase in aorta-to-IVC ratio increased the odds of some dehydration by 1.8 (95% CI: 1.4, 2.3) and severe dehydration by 1.6 (95% CI: 1.2, 2.1). After controlling for age, sex, and malnutrition status (MUAC), aorta to IVC ratio remained an independent predictor of both some dehydration and severe dehydration, with odds ratios of 1.5 (95% CI: 1.2, 1.9) and 1.4 (95% CI: 1.1, 1.9), respectively.

**Fig 2:**
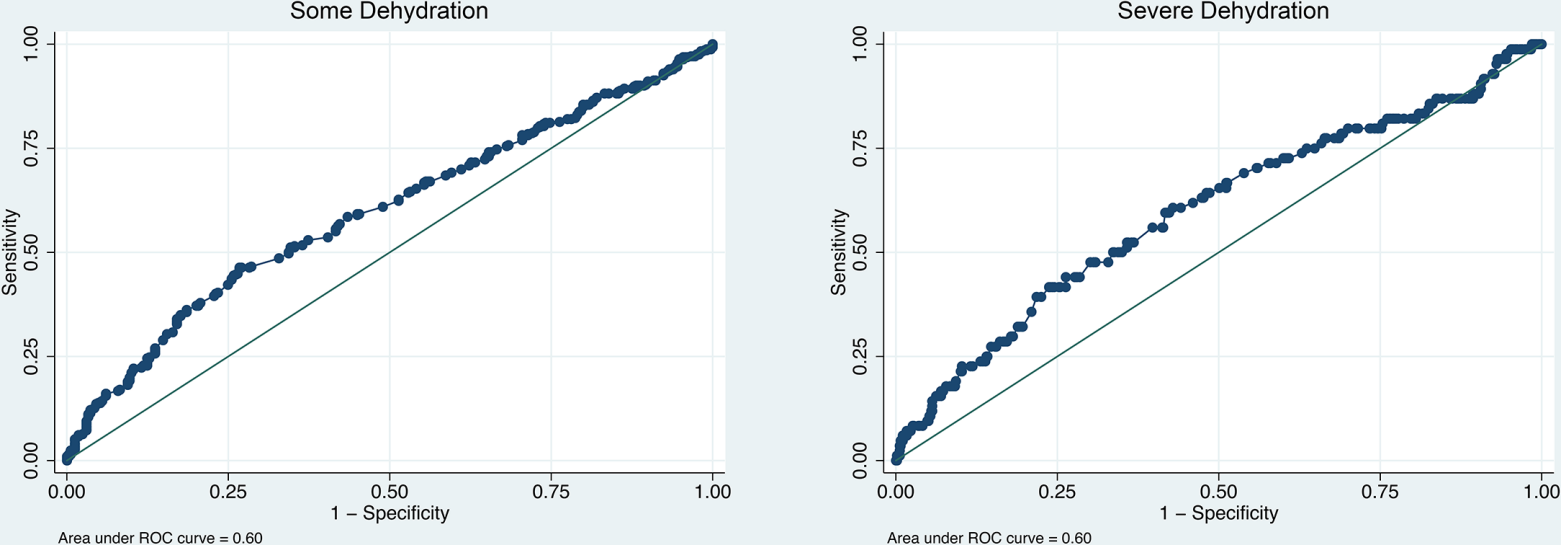
ROC curves of Aorta-to-IVC ratio for predicting dehydration.

The overall accuracy of aorta-to-IVC ratio was assessed using the area under its ROC curve (AUC) for the prediction of both some dehydration and severe dehydration. The aorta-to-IVC ratio had an overall AUC of 0.60 (95% CI: 0.56–0.64) for the prediction of some dehydration and 0.60 (95% CI: 0.53, 0.67) for the prediction of severe dehydration (Fig 2). Excluding exams marked as poor quality or those for which the research nurse had low confidence only marginally improved the overall AUCs for both severe and some dehydration (data not shown). Similarly, excluding children with malnutrition or those under 12 months of age did not impact the AUCs.

An aorta-to-IVC ratio of two or greater had a sensitivity of 67%, specificity of 49%, negative predictive value of 92%, and positive predictive value of 14% for the prediction of severe dehydration. The positive likelihood ratio for the prediction of severe dehydration was 1.3 (95% CI: 1.1, 1.5) and the negative likelihood ratio was 0.68 (95% CI: 0.50, 0.93).

### Repeat Measures

**Fig 3:**
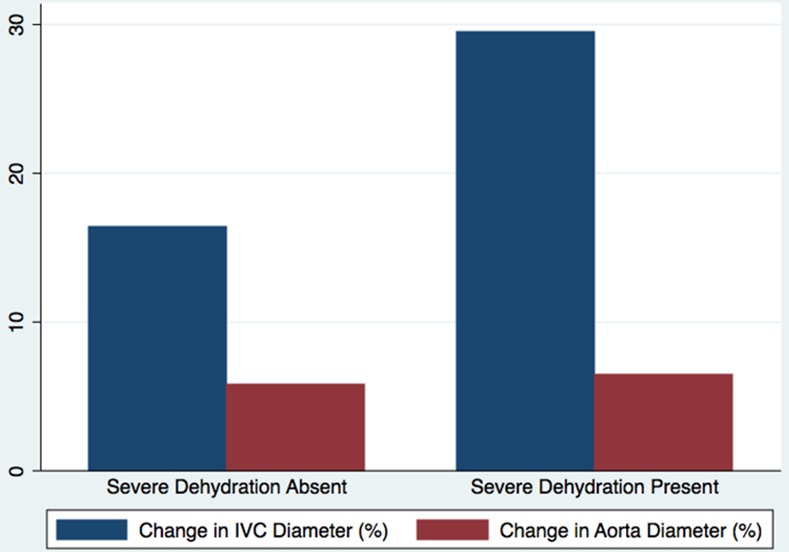
Change in IVC and Aorta diameter with Rehydration.

Of the 750 subjects with interpretable initial ultrasound exams, 289 were randomly selected for repeat ultrasonography after resuscitation to a stable weight. Overall, the mean IVC diameter increased by 18% between the admission and repeat ultrasound exam, while the mean aorta diameter increased by just 6%, a difference of 12% (95% CI: 6%, 17%). The mean change in IVC diameter was greater in subjects with severe dehydration (33%) than those without severe dehydration (17%), with a mean difference of 16% (95% CI: 1%, 32%) in the magnitude of IVC diameter change with rehydration between groups (Fig 3). Conversely, there was no difference in the magnitude of aorta diameter change with rehydration between patients with severe dehydration (6%) and those without severe dehydration (6%), with a mean difference of 0% (95% CI: -9%, 9%) between groups (Fig 3).

## Discussion

Our study sought to determine whether ultrasound of the aorta-to-IVC ratio could be used to predict the severity of dehydration in a population of children with acute diarrhea when used by nurses in a resource-limited setting. We found the aorta-to-IVC ratio was a statistically significant predictor of dehydration in our study population. However, the test characteristics of aorta-to-IVC ratio for predicting both some dehydration and severe dehydration in our study were poor. While we did find a clear increase in the IVC diameter with fluid resuscitation, and noted that the increase in IVC diameter was twice as great among children with severe dehydration as compared to those without severe dehydration, the discriminatory power of the aorta-to-IVC ratio was simply not strong enough for this test to be used in isolation by providers in a resource-limited setting to determine treatment for children with diarrhea.

Our findings contradict those of earlier, smaller studies, which have evaluated IVC diameter as a predictor of volume status in children. In a study of 73 children under age 15 with acute diarrhea at three rural hospitals in Rwanda, aorta-to-IVC ratio had an AUC of 0.76 (95% CI: 0.62, 0.90) for the prediction of severe dehydration, with a sensitivity of 93% and specificity of 59% [11]. In a study of 112 patients under 18 years of age conducted at a United States pediatric referral hospital, the IVC-to-aorta ratio predicted significant (>5%) dehydration, with an AUC of 0.73 (95% CI: 0.61, 0.84) and a sensitivity of 86% and specificity of 51% at the best cut-point of 0.8 [12]. A similar study of 113 patients conducted at another United States pediatric referral center identified an AUC for the IVC-to-aorta ratio of 0.72 (95% CI: 0.53, 0.91) for the prediction of significant dehydration, though the sensitivity and specificity of IVC-to-aorta ratio at the same cut-point of 0.8 were 67% and 71%, respectively [14]. Finally, a study of 51 patients under 21 years of age conducted in a pediatric intensive care unit in the United States found that the IVC-to-aorta ratio did not correlate with the central venous pressure, though a large percentage of the children in their study were mechanically ventilated [15].

There are several important differences between our study and previous ones in the literature that might explain the differences in our results. First, our study was much larger than prior ones, with a sample size greater than all prior studies combined, allowing for tight confidence intervals around our AUC. The three prior studies all had wide confidence intervals around their reported AUC that overlapped or nearly overlapped with our own AUC of 0.60, suggesting that the difference between our results and prior results may not be statistically meaningful.

Second, our study only enrolled children under five years of age, as this is the population with the highest risk of morbidity and mortality from acute diarrhea, in which accurate assessment of dehydration status is most likely to impact clinical outcomes. All prior IVC studies included older children as well, up to 15–21 years of age, which may affect the accuracy of aorta-to-IVC ratio as a predictor of dehydration. Children under five are less likely to lie still and more likely to cry during an ultrasound exam, which may make it more difficult to get accurate measurements. Crying may increase intra-thoracic and/or intra-abdominal pressure, skewing the measurement of IVC diameter. Younger children also have smaller IVCs, which may be more difficult to identify and measure accurately with a standard low frequency abdominal probe.

Finally, previous studies used physicians with some degree of prior ultrasound training to conduct the IVC and aorta measurements. Our study used clinical nurses who were given an intensive didactic and practical training, followed by ongoing feedback over the course of the study. The goal of this study was to develop new tools for assessing dehydration in children that could be used by frontline providers in resource-limited settings. Given that the majority of the 133 million outpatient and inpatient visits each year for acute diarrhea in children are attended to primarily by nurses working in resource-limited settings, we intentionally chose to use local, general practice nurses to perform all study procedures. Though our quality assurance review performed by ultrasound-fellowship trained physicians found the research nurses were able to identify and measure the correct vessels at least 76% of the time, misidentification of the IVC by operators with limited prior ultrasound experience may have accounted for the lower accuracy of aorta-to-IVC ratio in our study compared to previous ones. Indeed, prior research has found that ultrasound of the IVC performed by nurses correlates only moderately well with results obtained from ultrasound experts [16].

Our study suggests that ultrasound of the aorta-to-IVC ratio should not be used as the sole predictor of dehydration status in children presenting with acute diarrhea, especially when performed by novice sonographers or in young children. Given the clear increase in IVC size with hydration in children with significant volume deficits on arrival, multiple IVC ultrasounds performed on the same child over time may still be a helpful adjunct to guide resuscitation. Future research should determine whether ultrasound used in combination with clinical exam signs may better predict pediatric dehydration than either ultrasound or clinical exam alone. Finally, other ultrasound measurements of dehydration status, such as bladder ultrasound and carotid flow time, have shown promise in preliminary research, and their utility in the diagnosis of pediatric dehydration warrants further investigation in resource-limited settings [17, 18].

### Limitations

Our study population is not representative of all of children in the world with diarrhea, most of which never present to a health facility for clinical care.^1,2^ However, our study population is representative of children with acute diarrhea who present for medical care in resource-limited settings, and this is the population for which a dehydration assessment tool would be most relevant. Since all clinical services are free, icddr,b sees children from across the socioeconomic spectrum. About 90% of the children presenting to the rehydration unit at icddr,b arrive directly from home, with only 10% referred from other health facilities.

While the best physiologic criterion standard for dehydration remains the percent difference between pre-illness and admission weight, accurate pre-illness weights are rarely available for children in resource-limited settings. Instead we used the percent weight change with rehydration as the criterion standard for percent dehydration in our study, which correlates almost perfectly with percent volume loss and has been used in nearly all prior studies of dehydration in children [5,10]. Additionally, not all patients achieved a stable weight prior to discharge, allowing for the calculation of their percent dehydration. Although many patients returned later for repeat weight assessments, a total of 63 patients were lost to follow up and could not be included in our analysis. However, this represented a small fraction (7%) of the total sample size (n = 850), and there were no significant differences in baseline demographic and clinical variables between those patients included and excluded from our analysis.

## Conclusions

Point-of-care ultrasound of aorta-to-IVC ratio, though statistically associated with volume status in children presenting with acute diarrhea, was not accurate enough to be used as an independent screening tool for severe dehydration when used by nurses in a resource-limited setting to assess children under the age of five. Future research should focus on whether IVC ultrasound may be a more accurate predictor of dehydration when performed in older children or adults, when used in combination with clinical signs, or when evaluated by more experienced operators. Other ultrasound measures of dehydration status, including bladder ultrasound and carotid flow time, warrant further investigation as measures of dehydration in the pediatric population.

## Supporting Information

S1 File Ultrasound Protocol. (DOCX)

S2 File Minimal Dataset. (XLS)
