# Supplementary material for: Accuracy of Inferior Vena Cava Ultrasound for Predicting Dehydration in Children with Acute Diarrhea in Resource-Limited Settings
Source: PLoS One. 2016 Jan 14;11(1):e0146859. doi: 10.1371/journal.pone.0146859 (PMC4713074; doi:10.1371/journal.pone.0146859)
Supplement: S1 File — (DOCX) [file pone.0146859.s001.docx]

Ultrasound Protocol

1. Place the Sonosite M-Turbo ultrasound machine on top of the Study Trolley, next to the subject’s bed, lift the ultrasound machine cover, and turn the machine ON.
2. Press the “PATIENT” button and enter the last name and study ID number, then press “DONE.”
3. Lay the child flat on the bed or trolley. Small children can lie in their mother’s lap in order to keep them calm, but **the child must be parallel to the ground**.
4. Use the standard low frequency abdominal ultrasound probe for most subjects. For very young infants (less than 3 months), the high frequency vascular probe may achieve better resolution of the IVC and aorta.
5. Set the depth to 8cm for infants (< 1 year) and 10-12 cm for children 1-5 years. (In general, it is best to do as much as you can before placing the probe on the subject. Many of these children are already irritable, and even though the ultrasound exam is painless, they will usually cry when you place the probe on their abdomen.)
6. Place the probe in the subject’s midline, just inferior to the xyphoid process. The probe should be oriented in the transverse plane, with the marker pointed to the subject’s right.
7. In this position, you should see the left lobe of the liver at the top of the screen and the vertebral shadow at the bottom (see Figure 1 below). The aorta will be a circular structure just above the vertebral shadow. If you have trouble finding it try using color mode to find the aortic pulsations. The inferior vena cava (IVC) will be just to the left of the aorta on the screen. It will be roughly circular, but may also be oval shaped depending on the degree of dehydration and the exact angle of the probe.
8. If your view is obstructed by bowel gas, you can press down GENTLY to disperse the bowel gas. The subjects are all small and thin, so excessive pressure is never necessary to see the aorta and IVC. You can also try moving the probe a few centimeters to the subject’s right so that you are viewing through the liver.
9. As soon as you can visualize the aorta and IVC in the transverse plane, save a short video loop (6 seconds).
10. As soon as the loop is saved, FREEZE the image on the screen, and then remove the probe from the subject’s abdomen.
11. Scroll backwards one image at a time using the arrow (🡨 and 🡪) buttons until you find the aorta at its maximal width (in systole). Use the calipers to measure the anterior-posterior aorta diameter (inner wall to inner wall for the purpose of this study) and save the image (see Figure 2 below).


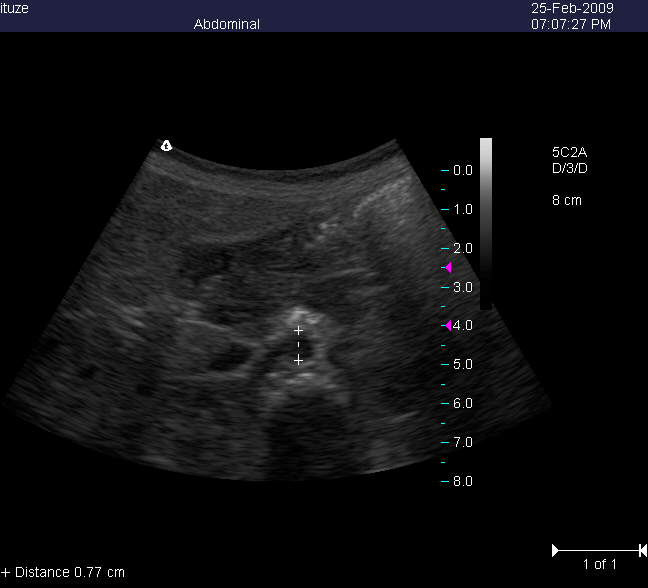


**Figure 1**

1. Scroll back a few more frames until you find the IVC at its largest width (in expiration). Use the calipers to measure the anterior-posterior IVC diameter (inner wall to inner wall) and save the image (see Figure 4 below).


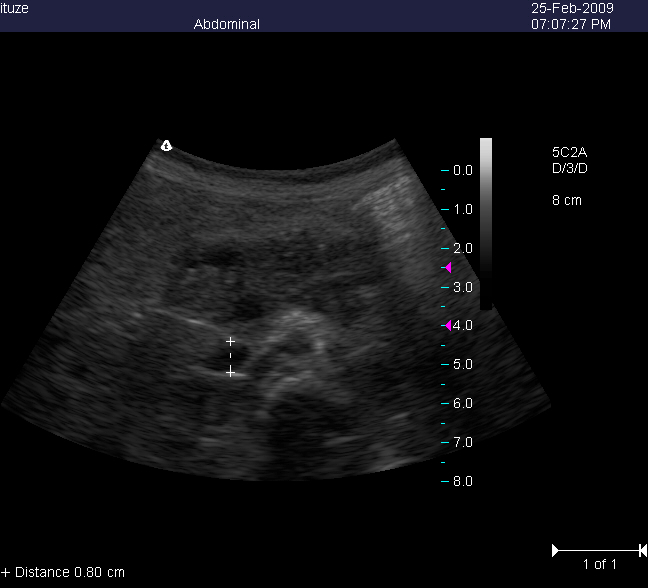


**Figure 2**

1. Press REVIEW to ensure that you have saved exactly one 6-second video loop, one still image of the aorta with a measured diameter, and one still image of the IVC with a measured diameter.
2. Record the IVC and aorta diameters in centimeters. Be sure to place a 0 in front of the decimal place if appropriate. Fill in the first name of the researcher performing the exam and the time the exam was performed.
3. Enter the approximate length of time to perform the ultrasound. The research nurse or physician performing the ultrasound should enter their own personal assessment of the image quality: Good if the image was clear, Poor if it was not clear, and Uninterpretable if they were not able to measure the IVC or aorta.
4. The research nurse or physician should enter their personal confidence in the scan (how certain they were that they measured the correct vessels and the correct length) on a scale from 1 to 5, where 1 means not at all confident (uncertain) and 5 means very confident (certain).
5. Turn off the M-Turbo ultrasound machine and close the cover.
6. Wipe off the ultrasound probe with the disinfectant spray and carefully put it away in the Study Trolley.
